# The Optimal Ethnic-Specific Waist-Circumference Cut-Off Points of Metabolic Syndrome among Low-Income Rural Uyghur Adults in Far Western China and Implications in Preventive Public Health

**DOI:** 10.3390/ijerph14020158

**Published:** 2017-02-08

**Authors:** Jia He, Rulin Ma, Jiaming Liu, Mei Zhang, Yusong Ding, Heng Guo, Lati Mu, Jingyu Zhang, Bin Wei, Yizhong Yan, Jiaolong Ma, Hongrui Pang, Shugang Li, Shuxia Guo

**Affiliations:** Department of Public Health and Key Laboratory of Xinjiang Endemic and Ethnic Diseases of the Ministry of Education, School of Medicine, Shihezi University, Shihezi 832002, China; hejia123.shihezi@163.com (J.H.); marulin@126.com (R.M.); liujiaming@shzu.edu.cn (J.L.); zmberry@foxmail.com (M.Z.); 13399931625@163.com (Y.D.); guoheng@shzu.edu.cn (H.G.); murat08123@163.com (L.M.); yfyxxzjy@126.com (J.Z.); weibindavid@gmail.com (B.W.); erniu19880215@sina.com (Y.Y.); jiaojiaolong881202@163.com (J.M.); 15909938133@163.com (H.P.); lishugang@ymail.com (S.L.)

**Keywords:** ethnic-specific, metabolic syndrome, cut-off points of waist circumference, low-income rural setting, Uyghur, public health

## Abstract

*Background:* Metabolic syndrome is pandemic; however, the cut-off values for waist circumference (WC) vary widely depending on the ethnic groups studied and the criteria applied for WC measurement. Previous studies for defining optimal WC cut-off points included high-income and urban settings, and did not cover low-income, rural settings, especially for ethnic minorities. This study aimed at defining optimal ethnic-specific WC cut-off points in a low-income, rural population comprising the largest inhabitant minority group residing in far Western China. *Methods*: Questionnaire-based surveys, physical examinations, and blood testing of 3542 individuals were conducted in 2010, using a stratified cluster random sampling method in rural Uyghur residents (≥18 years old) from 12 villages in Xinjiang, China, approximately 4407 km away from the capital city, Beijing. Metabolic syndrome was defined according to the International Diabetes Federation (IDF) criteria. Optimal, ethnic-specific WC cut-off values for diagnosing metabolic syndrome were determined using receiver operator characteristic (ROC) curve analysis. *Results*: As WC increased, there was a significant, increasing trend of detection and risk in rural Uyghur adults, regardless of the presence of ≥1 or ≥2 components of metabolic syndrome by IDF criteria. The optimal ethnic-specific WC cut-off point to predict the presence of metabolic syndrome was 85 cm for men and 82 cm for women. With these cut-off points, the prevalence rates of metabolic syndrome among men, women, and overall population in Uyghur adults were 19.5%, 23.0%, and 21.3%, respectively. *Conclusions*: We report a high prevalence of metabolic syndrome, especially in women, among rural Uyghurs in Western China. A WC cut-off of 85 cm in men and 82 cm in women was the best predictor of metabolic syndrome in this population. Because of the cost-effectiveness in measuring WC, we recommend that these WC cut-off points be integrated into local preventive policies for public health as the primary screening criteria for metabolic syndrome and related diseases among low-income, rural minorities.

## 1. Introduction

Metabolic syndrome is characterized by clustering of metabolic abnormalities such as central obesity, hypertension, dyslipidemia, and glucose intolerance. Together, these abnormalities increase the risk of diabetes mellitus, cardiovascular disease, stroke, and overall mortality [1,2]. Of these risk factors, central obesity is considered as the most important risk factor [3,4]. As an indirect measure of visceral fat, waist circumference (WC) is an easy, cost-effective, and non-invasive metric useful for identification of central obesity and, in turn, may be an effective predictor of the risk of metabolic syndrome [5,6]. However, ethnic and racial variation among populations from different regions warrants different cut-off points to diagnose metabolic syndrome [7,8].

Uyghurs are the largest minority group in the Xinjiang Uyghur Autonomous Region, in far Western China. Due to low-income and limited resources in public health combined with poor transportation availability, there is a lack of studies analyzing local public health needs, including prevalence of metabolic syndrome and related diseases. Uyghurs have a mixture of 60% European ancestry and 40% East Asian ancestry [9]. Due to their specific genetic background, religion, culture, lifestyle, and diet, research on the optimal ethnic-specific WC cut-off points of metabolic syndrome for Uyghurs is essential and may reveal valuable information for determining appropriate policies for preventive public health for the residents of Xinjiang.

Studies investigating the WC cut-off points of metabolic syndrome for Uyghurs are rare, and have reported inconsistent results [10,11]. In addition, the data for these studies were gathered mainly from high-income and urban settings, and lacked corresponding data from large samples in low-income, rural settings. This study is a cross-sectional epidemiologic study among rural Uyghur adults residing in far Western China, which allowed for the determination of the prevalence of metabolic syndrome using the International Diabetes Federation (IDF) criteria, in addition to determining the optimal ethnic-specific WC cut-off points for prediction of the presence of metabolic syndrome in this population. Our findings may have important implications in preventive public health for medically underserved Muslim minorities.

## 2. Methods

### 2.1. Ethics Statement

The Institutional Ethics Review Board (IERB) at the First Affiliated Hospital of Shihezi University School of Medicine approved this study (IERB No. SHZ2010LL01). Standard university hospital guidelines including those for informed consent, voluntary participation, confidentiality, and anonymity, were followed. All participants gave written informed consent before the study began.

### 2.2. Settings and Study Population

The survey was conducted from March to April 2010 in the Kashi prefectures, about 4407 km (2739 miles) away from Beijing. In this region, approximately 98% of the population is comprised of minority Muslim Uyghurs. A multistage (prefecture-county-township-village) stratified cluster random sampling method was used to select participants. Initially, we chose the representative prefecture (Kashi) according to the geographical distributions of minority populations in Xinjiang, a province in Northwestern China. We then randomly selected one county in this prefecture, and one township from the county (Jiangbazi Township in Jiashi County). Then, a stratified sampling method was used to draw corresponding villages in the township (12 villages in Jiangbazi Township). We interviewed local Uyghurs aged 18 years or older residing in the village for at least 6 months. We successfully interviewed a total of 3542 individuals with an overall response rate of 88.6%.

### 2.3. Questionnaire Survey

A self-developed questionnaire was utilized to collect detailed information from all respondents during face-to-face interviews. The questionnaire consisted of demographic information (such as age, sex, education level, and marital status, etc.) and personal lifestyle information (such as smoking, alcohol intake and dietary habits, etc.).

### 2.4. Physical Examinations

Anthropometric measurements of WC were obtained using a standard protocol for participants during the interview and physical examinations [12]. WC was defined as the midpoint between the lower rib and upper margin of the iliac crest, measured by a ruler tape with an insertion buckle at one end. WC was measured to the nearest 0.1 cm. Blood pressure was measured three times using a digital device (Omron M6, Omron, Kyoto, Japan). Appropriate cuff sizes were used, and measurements were made while the participants were seated and resting with a break of several minutes between measurements. The first blood pressure measurement was discarded, and the second and third measurements were then averaged.

### 2.5. Biochemical Measurements

Fasting blood samples were obtained from an antecubital vein with minimum stasis. Plasma and serum samples were obtained by centrifugation at 200× *g* for 20 min at 4 °C, and aliquots were stored at −70 °C until assayed. High-density lipoprotein cholesterol (HDL-C), triglyceride (TG), and fasting plasma glucose (FPG) concentrations were measured using an auto-analyzer (Randox Daytona Clinical Analyzer Randox Laboratories, Crumlin, UK) and enzymatic methods.

### 2.6. Definitions

Metabolic syndrome was diagnosed using the new harmonized guidelines of the International Diabetes Federation [13] as central obesity (WC ≥ 90 cm in men and ≥80 cm in women) with two or more of the following conditions: (1) serum TG level ≥ 1.7 mmol/L, or receiving treatment for this lipid abnormality; (2) HDL-C level <1.03 mmol/L in men and <1.29 mmol/L in women, or receiving treatment for this lipid abnormality; (3) systolic blood pressure (SBP) ≥ 130 mmHg and/or diastolic blood pressure (DBP) ≥ 85 mmHg, or receiving treatment for previously diagnosed hypertension; and (4) FPG ≥ 100 mg/dL (5.6 mmol/L), or previously diagnosed type 2 diabetes.

### 2.7. Statistical Analysis

Data were analyzed using the Statistical Package for Social Sciences version 20 (SPSS Inc., Chicago, IL, USA) software. Continuous variables were presented as mean ± standard deviation and analyzed using the *t*-test. Categorical variables were expressed as numbers or percentages and analyzed using the chi-squared and trend tests. Logistic regression was performed to determine if there was a significant increase in disease risk at the optimal waist cut point by calculating the odds ratio (OR) with associated 95% confidence interval (95% CI) for metabolic syndrome. Optimal ethnic-specific WC cut-off values for diagnosing metabolic syndrome were determined using receiver operator characteristic (ROC) curve analysis with the Youden index [maximum (sensitivity + specificity − 1)] [14]. First, we created dichotomous variables for each cut-off value of WC from 75 cm to 105 cm. Then, we calculated the sensitivity and specificity of each WC cut-off point to detect at least two other components of metabolic syndrome using the formulas of the diagnostic tests. Finally, we calculated the Youden index. A cut-off value with the maximum Youden index of the ROC curve was defined as the optimal WC cut-off point to diagnose metabolic syndrome. All statistical tests were two-sided, and differences were considered statistically significant when the *p* value < 0.05. 

## 3. Results

### 3.1. Characteristics of the Study Populations

The 3542 study participants included Uyghur adults, of which 1728 were men (48.8%) with an average age of 42.58 ± 16.18 years, and 1814 were women (51.2%) with an average age of 43.13 ± 15.82 years). Baseline characteristics are listed in Table 1. All of the characteristics shown in the table differed significantly between the two sexes, except the average age (*p* < 0.05 for each comparison). Women had a higher average HDL-C level while the values of other characteristics were lower than the corresponding values for men (Table 1).

### 3.2. Relationship between WC and the Components of Metabolic Syndrome Based on IDF Criteria

Table 2 shows the detection and calculated risk (odds ratio (95% confidence intervals)) for the components of metabolic syndrome in men and women based on the IDF criteria for the studied WC classifications. As WC increased, there was a significant increasing trend in the detection of metabolic syndrome in both men and women, regardless of whether ≥1 or ≥2 components of metabolic syndrome (except WC) were considered, after adjustment for age, smoking, and drinking [Men (≥1 components of metabolic syndrome: *χ*^2^*_trend_* = 64.242, *p* < 0.001; ≥2 components of metabolic syndrome: *χ*^2^*_trend_* = 94.014, *p* < 0.001). Women (≥1 components of metabolic syndrome: *χ*^2^*_trend_* = 46.283, *p* < 0.001; ≥2 components of metabolic syndrome: *χ*^2^*_trend_* = 129.871, *p* < 0.001)]. Of note, the risk associated with each of the components of metabolic syndrome followed the same trend as that of the number of cases of metabolic syndrome detected.

### 3.3. The Optimal Ethnic-Specific WC Cut-Off Values of Metabolic Syndrome among Low-Income Rural Uyghur Adults

**Figure 1:**
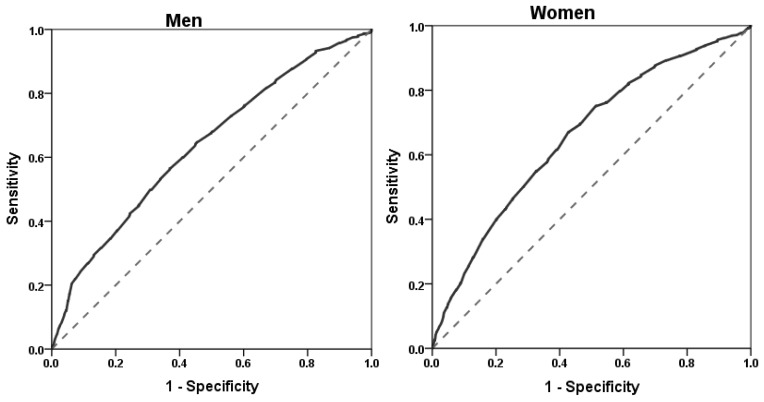
Receiver operator characteristic (ROC) curves for waist circumference (WC) to predict the presence of at least two other components of the metabolic syndrome, as defined by the IDF, in men and women. Area under the ROC curve: 0.636 in men and 0.658 in women. WC cut-off point: 85 cm in men (sensitivity 60.0%, specificity 59.3%) and 82 cm in women (sensitivity 67.0%, specificity 57.3%).

According to the IDF criteria, ROC curve analysis was used to determine the optimal ethnic-specific cut-off points of WC for predicting at least two components of metabolic syndrome (Table 3, Figure 1). Among men, WC at a cut-off value of 85 cm resulted in the highest Youden index (0.193) with a corresponding sensitivity of 60.0% and specificity of 59.3% (Area under the ROC curve: 0.636, standard error: 0.014, *p* < 0.001, 95% confidence intervals: 0.608–0.664). At the traditional cut-off value of 90 cm of WC for men, the sensitivity dropped to 41.5%, and specificity increased slightly to 75.8%. Similarly, among women, WC at a cut-off point of 82 cm resulted in the highest Youden index (0.243) with the corresponding sensitivity and specificity of 67.0% and 57.3%, respectively (Area under the ROC curve: 0.658, standard error: 0.013, *p* < 0.001, 95% confidence intervals: 0.631–0.684). At the traditional cut-off point of 80 cm WC among women, the sensitivity sharply increased to 75.2%, but this occurred at the cost of a significant drop in the specificity from 57.3% to 48.7% (Table 3).

### 3.4. Prevalence Rates of Metabolic Syndrome Using the Ethnic-Specific and Traditional WC Cut-Off Values by IDF Criteria

**Figure 2:**
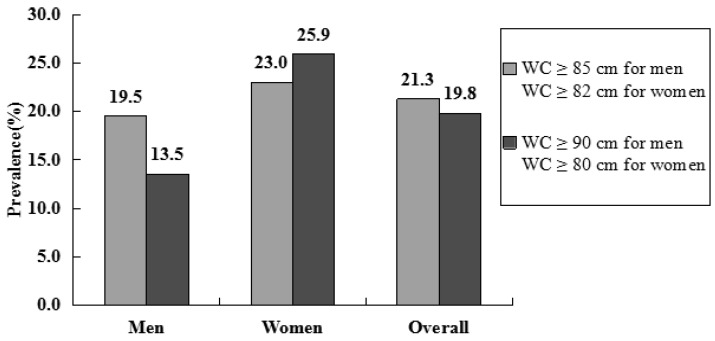
Prevalence rates of metabolic syndrome in rural Uyghur adult using the ethnic-specific and traditional waist circumference (WC) cut-off values by IDF criteria. Ethnic-specific WC cut-off values: WC ≥ 85 cm for men, and ≥82 cm for women; traditional WC cut-off values: WC ≥ 90 cm for men, and ≥80 cm for women.

The prevalence of metabolic syndrome among men and women (as shown in Figure 2), and the overall prevalences in Uyghur adults were 19.5%, 23.0%, and 21.3%, respectively, using the ethnic-specific cut-off points of WC (WC ≥ 85 cm for men, and ≥82 cm for women). The corresponding prevalence rates with the traditional cut-off points (WC ≥90 cm for men, and ≥80 cm for women) were 13.5%, 25.9%, and 19.8%, respectively. The prevalence rate among men and the overall prevalence, according to the ethnic-specific cut-off points of WC, were higher than those according to the traditional cut-off points (*p* < 0.001). Alternatively, the prevalence of metabolic syndrome in women was lower with ethnic-specific cut-off point than when using traditional cut-off points (*p* < 0.001). Finally, for both the ethnic-specific and traditional cut-off points, the prevalence of metabolic syndrome in women was higher than that in men (*p* < 0.05 for both comparisons).

## 4. Discussion

The findings of the present study demonstrate the superlative discriminating values of common anthropometric parameters for metabolic syndrome among Uyghur adults. While some previous studies were performed among national minorities, to the best of our knowledge this is the first study that was performed exclusively in low-income rural Uyghur adults who live in far Western China. 

There is controversy about the proper anthropometric values relative to ethnicity, genetic background, sexes, and sociocultural context. Beydoun et al. showed that WC is among the most powerful tools for predicting metabolic syndrome and that the optimal cut-off values for various indices, including WC, may differ by sex and race [3]. Beydoun’s suggestion is consistent with other reports [8,15,16,17].

The IDF definition of metabolic syndrome requires that abdominal obesity should be a precondition for the diagnosis of metabolic syndrome, and underline WC as a simple screening tool [13]. For those of Asian ethnicity, the IDF-specified cut-off value of WC for abdominal obesity is ≥90 cm in men and ≥80 cm in women. Some researchers have also suggested WC cut-off value for abdominal obesity in Chinese individuals as ≥90 cm for men and ≥85 cm for women [18,19]. If the rate of metabolic syndrome in Chinese population is assessed using the IDF definition, there is a risk of bias due to the cut-off value for abdominal obesity in women not being appropriate for Chinese women.

As shown in Table 4, the optimal cut-off point of WC for the diagnosis of metabolic syndrome is distinct in different Asian countries. In the present study, the optimal WC cut-off value was 85 cm and 82 cm for identifying metabolic syndrome in men and women, respectively, with their highest Youden indices of 0.193 and 0.243, respectively. The best WC cut-off value for men is higher than in Japan [20] and Korea [21], but lower than in Singapore, India, Iran, and in the Han population in China [22,23,24,25]. The WC cut-off value for women is lower in this population than in Iran and China as a whole, but higher than in other countries.

The sex-related difference in the prevalence of metabolic syndrome in this study (19.5% in men and 23.0% in women) is similar to that observed in previous studies from Iran (10.7% in men and 35.1% in women) [26], Korea (48.1% in men and 64.3% in women) [27], and other regions [28,29]. The high rate of obesity and other factors, such as physical activity and dietary intake, may be associated with the high prevalence of metabolic syndrome in women. Our previous reports found that women had a higher prevalence of obesity in rural Chinese Uyghur adults [30]. This study confirms that women should be prioritized when considering preventive public health in far Western China, and that the simple addition of measuring WC may have important implications in national screening programs. 

The incidence of individual components of metabolic syndrome in rural Uyghur adults found that low HDL cholesterol was most common individual component in women (89.4%) and high blood pressure was most common individual component in men (79.5%). Alternatively, the rarest component was high FPG in both women and men (10.6% and 14.5%, respectively). This means that the high rate of metabolic syndrome in rural Uyghur adults is likely driven by the high prevalence of elevated blood pressure in men and low HDL serum concentration in women. Other surveys in different populations have also discovered a high incidence of low HDL cholesterol, even in individuals without obesity and hypertriglyceridemia, supporting an ethnic predisposition to this type of dyslipidemia [31,32,33]. These findings may indicate that, if the current trend continues, this population will have higher risk for coronary artery disease. Our research group has already begun genetic studies on this potential ethnic predisposition among low-income rural Uyghur adults [34,35], but the specific mechanism remains to be elucidated. 

Recent studies have shown that individuals of Asian ethnicity have less lean muscle mass and more visceral fat mass at a lower BMI (Body Mass Index) and WC than is observed in Western populations [25,36]. An important conclusion of this study is that the WC cut-off currently used for the diagnosis of metabolic syndrome in Uyghur adults (90 cm for men, 80 cm for women) is not suitable and will result in a lower estimation of the prevalence of metabolic syndrome. Using the new WC cut-off point in this study (85 cm for men, 82 cm for women), the prevalence of metabolic syndrome is 21.3%, as compared to 19.8% when using the traditional cut-off point. This underestimation of the prevalence of metabolic syndrome will reduce the number of subjects requiring therapy, and may have a negative influence for any national intervention programs aimed at subjects with health promotion. In low-income regions where financial resources and infrastructure are constrained, such as the rural regions in which Uyghur adults reside in far Western China, more than 92% of individuals live on $1.00 USD per day or less, a sharp contrast to the 2005 national average of 15.9% of people living on $1.00 USD per day [37,38] (USD $1 = CNY¥6.623, 31 December 2010, Bank of China). Per capita income of $0.50 USD per day (USD $181 per year) was the Chinese rural poverty line in 2010 and before. Therefore, we have used this as the definition of the “low income” in this study. Furthermore, many live too far from the city to seek medical advice due to the inconvenience of travel and the relative deficiency of medical service resources. Therefore, WC as a simple and cost-effective screening indicator is a perfect choice for early detection of metabolic syndrome in Uyghur adults.

This study was cross-sectional in design, so the optimal cut-off point for WC needs to be tested through longitudinal studies. Furthermore, in both in men and women, the sensitivity and specificity of the WC cut-off value were not high (60.0% and 59.3% in men, 67.0% and 57.3% in women, respectively). However, for metabolic syndrome, the most common chronic, non-communicable disease of the low-income rural Uyghur adults, accurate diagnoses is more important than high sensitivity or specificity, because of the lack of health awareness and medical resources for this low-income minority. Considering the role of public health, we chose 85 cm for men and 82 cm for women as the optimal ethnic-specific WC cut-off values in the present study. According to the results of members of our research group [39,40], this representative sample with valid weights for standardization and the large sample size can help us to infer the distribution and epidemiology of metabolic syndrome, and its components in low-income rural Uyghur adults as well as the region as a whole.

Optimal ethnic-specific WC cut-off points appear to be useful as a screening tool that provides benefits not only in the detection of obesity, but also in assessing the risk of other related diseases such as diabetes and cardiovascular disease. In this context, our observations provide up-to-date, evidence-based data that can be used for public health policies in low-income populations in Asian countries, particularly among medically underserved Muslim minorities, aiding in the prevention and early control of modifiable factors in this region. 

## 5. Conclusions

We report a high prevalence of metabolic syndrome, especially in women, among rural Uyghurs in Western China. A WC cut-off of 85 cm in men and 82 cm in women was the best predictor of metabolic syndrome in this population. Because of the cost-effectiveness in measuring WC, we recommend that these WC cut-off points be integrated into local preventive policies for public health as the primary screening criteria for metabolic syndrome and related diseases among low-income, rural minorities.

## Figures and Tables

**Table 1:** Characteristics of the study population.

Characteristic	Men	Women	Total
*n*	1728	1814	3542
Age (years) ^#^	42.58 ± 16.18	43.13 ± 15.82	42.86 ± 15.99
Age-group (years) ^##^			
18–24	254 (14.7)	248 (13.7)	502 (14.2)
25–34	373 (21.6)	369 (20.3)	742 (20.9)
35–44	391 (22.6)	420 (23.2)	811 (22.9)
45–54	288 (16.7)	315 (17.4)	603 (17.0)
55–64	228 (13.2)	269 (14.8)	497 (14.0)
≥65	194 (11.2)	193 (10.6)	387 (10.9)
WC (cm) ^#^	85.15 ± 9.50 *	82.77 ± 10.93	83.93 ± 10.32
SBP (mmHg) ^#^	126.84 ± 18.96 *	122.87 ± 21.55	124.81 ± 20.42
DBP (mmHg) ^#^	79.95 ± 12.30 *	76.94 ± 13.30	78.41 ± 12.91
TG (mmol/L) ^#^	1.37 ± 1.20 *	1.19 ± 0.75	1.28 ± 1.00
HDL-C (mmol/L) ^#^	1.18 ± 0.62 *	1.28 ± 0.66	1.23 ± 0.64
FPG (mmol/L) ^#^	4.45 ± 1.10 *	4.33 ± 1.04	4.39 ± 1.07
High blood pressure (%) **	46.8 *	36.8	41.7
Low HDL-C (%) **	43.7 *	67.2	55.7
High TG (%) **	22.0 *	17.0	19.4
High FPG (%) **	6.5 *	4.6	5.5

WC: waist circumference; SBP: systolic blood pressure; DBP: diastolic blood pressure; TG: triglyceride; HDL-C: high-density lipoprotein cholesterol; FPG: fasting plasma glucose. ^#^ Data were expressed as mean ± standard deviation. ^##^ Data were expressed as *n* (%). * *p* < 0.05 versus women from the same ethnic group. ** Data were diagnosed using the new harmonized guidelines of the International Diabetes Federation (IDF).

**Table 2:** Relationship between waist circumference (WC) and the components of metabolic syndrome based on IDF criteria.

WC (cm)	*n*	One or More Components ^#^		Two or More Components ^#^	
		*n*	Risk *	*n*	Risk *
Men					
<80	486	322	1.00	103	1.00
80–84	431	319	1.38 (1.04–1.84)	124	1.45 (1.07–1.97)
85–89	295	224	1.49 (1.07–2.07)	103	1.88 (1.35–2.60)
90–94	235	193	2.03 (1.37–3.00)	83	1.80 (1.26–2.56)
95–99	137	118	2.80 (1.65–4.77)	66	3.12 (2.08–4.70)
100–104	85	82	11.48 (3.55–37.07)	50	4.57 (2.79–7.49)
≥105	59	53	3.84 (1.60–9.20)	35	4.69 (2.65–8.30)
*χ*^2^*_trend_*		64.242		94.014	
*p* value		<0.001		<0.001	
Women					
<75	441	324	1.00	80	1.00
75–79	293	240	1.56 (1.07–2.26)	75	1.34 (0.92–1.94)
80–84	351	282	1.34 (0.95–1.89)	127	2.25 (1.61–3.15)
85–89	278	228	1.46 (1.00–2.15)	109	2.26 (1.58–3.22)
90–94	188	168	2.58 (1.54–4.33)	89	3.20 (2.17–4.71)
95–99	130	116	2.51 (1.38–4.58)	65	3.49 (2.26–5.40)
≥100	133	125	4.70 (2.22–9.96)	79	5.05 (3.26–7.81)
*χ*^2^*_trend_*		46.283		129.871	
*p* value		<0.001		<0.001	

^#^ Components included: (1) serum TG level ≥1.7 mmol/L, or receiving treatment for this lipid abnormality; (2) HDL-C level <1.03 mmol/L in men and <1.29 mmol/L in women, or receiving treatment for this lipid abnormality; (3) SBP ≥ 130 mmHg and/or DBP ≥ 85 mmHg, or receiving treatment of previously diagnosed hypertension; and (4) FPG ≥ 100 mg/dL (5.6 mmol/L), or previously diagnosed type 2 diabetes. TG: triglyceride; HDL-C: high-density lipoprotein cholesterol; SBP: systolic blood pressure; DBP: diastolic blood pressure; FPG: fasting plasma glucose. * Risk was expressed as an odds ratio (95% confidence intervals) compared against the first group of waist circumference each gender, with adjustment for age, smoking, drinking.

**Table 3:** The optimal ethnic-specific waist circumference (WC) cut-off values to detect subjects with two or more components of metabolic syndrome based on IDF criteria.

Gender	WC (cm)	Sensitivity (%)	Specificity (%)	PPV (%)	NPV (%)	Youden Index
Men						
	≥80	81.7	32.9	37.1	78.8	0.146
	≥81	75.7	39.9	37.9	77.2	0.156
	≥82	72.9	44.0	38.7	77.0	0.169
	≥83	67.6	50.2	39.6	76.1	0.178
	≥84	63.7	55.2	40.7	75.8	0.189
	≥85	60.0	59.3	41.6	75.2	0.193
	≥86	56.1	63.1	42.4	74.8	0.192
	≥87	53.0	66.1	43.1	74.4	0.191
	≥88	49.8	69.2	44.0	74.0	0.190
	≥89	44.7	72.9	44.4	73.1	0.176
	≥90	41.5	75.8	45.3	72.8	0.173
	≥95	26.8	88.8	53.7	71.5	0.156
	≥100	15.1	94.9	59.0	69.8	0.100
	≥105	6.2	97.6	59.3	68.3	0.038
Women						
	≥75	87.2	30.3	39.6	81.9	0.175
	≥80	75.2	48.7	43.4	78.9	0.239
	≥81	69.4	53.6	44.0	77.0	0.230
	≥82	67.0	57.3	45.1	76.8	0.243
	≥83	61.5	61.1	45.3	75.2	0.226
	≥84	57.7	64.0	45.7	74.3	0.217
	≥85	54.8	67.5	46.9	74.0	0.223
	≥86	50.2	71.3	47.9	73.2	0.215
	≥87	46.2	74.9	49.1	72.6	0.211
	≥88	43.1	77.1	49.7	72.1	0.202
	≥89	39.9	79.9	51.0	71.7	0.198
	≥90	37.4	81.7	51.7	71.3	0.191
	≥95	23.1	90.0	54.8	69.1	0.131
	≥100	12.7	95.5	59.4	67.6	0.082

PPV: positive predictive value; NPV: negative predictive value.

**Table 4:** Reports on optimal cut-off point of waist circumference (WC) for the diagnosis of metabolic syndrome in Asian countries.

Country [Reference Number]	*n*	Prevalence of Metabolic Syndrome (%)	Cut-Off Point for Men (cm)	Cut-Off Point for Women (cm)
Japan [20]	5972	32.8 ^#^	84	80
Singapore [22]	4723	17.9 *	90	80
India [23]	640	29.9 *	90	80
Korea [21]	31,076	-	83	76
Iran [24]	5332	30.4 ^&^	89	86
China [25]	47,325	24.2 ^&^	90	85

Note: ^#^ = Metabolic syndrome was defined among men and women as WC in excess of 85 cm and 90 cm, respectively; * = Metabolic syndrome was defined among men and women as WC in excess of 102 cm and 88 cm, respectively; ^&^ = Metabolic syndrome was defined among men and women as WC in excess of 90 cm and 80 cm, respectively.
